# Comprehensive Gene Expression Analysis to Identify Differences and Similarities between Sex- and Stage-Stratified Melanoma Samples

**DOI:** 10.3390/cells11071099

**Published:** 2022-03-24

**Authors:** Eirini Chrysanthou, Emir Sehovic, Paola Ostano, Giovanna Chiorino

**Affiliations:** 1 Department of Life Sciences and Systems Biology, University of Turin, 10100 Turin, Italy; eirini.chrysanthou@unito.it (E.C.); emir.sehovic@unito.it (E.S.); 2 Cancer Genomics Lab, Fondazione Edo ed Elvo Tempia, 13900 Biella, Italy; paola.ostano@fondazionetempia.org

**Keywords:** melanoma, sexual dimorphism, gene expression, tumor stage

## Abstract

Overall lower incidence and better prognosis are observed in female melanoma patients compared to males. As sex and stage differences in the context of melanoma gene expression are understudied, we aim to highlight them through statistical analysis of melanoma gene expression datasets. Data from seven online datasets, including normal skin, commonly acquired nevi, and melanomas, were collected and analyzed. Sex/stage-related differences were assessed using statistical analyses on survival, gene expression, and its variability. Significantly better overall survival in females was observed in stage I, II but not in stage III. Gene expression variability was significantly different between stages and sexes. Specifically, we observed a significantly lower variability in genes expressed in normal skin and nevi in females compared to males, as well as in female stage I, II melanomas. However, in stage III, variability was lower in males. Similarly, class comparison showed that the gene expression differences between sexes are most notable in non-melanoma followed by early-stage-melanoma samples. Sexual dimorphism is an important aspect to consider for a holistic understanding of early-stage melanomas, not only from the tumor characteristics but also from the gene expression points of view.

## 1. Introduction

Sexual disparities in cancer have been consistently consolidated in literature, with growing evidence of sex affecting cancer incidence, overall survival, and treatment response [1]. These disparities, which are apparent across most non-reproductive tumor types, are due to the interplay between hormones, the immune system as well as genetic and epigenetic factors [2]. Despite the fact that sexual dimorphism in cancer extends to all aspects of oncogenicity, many transcriptomic and biomarker-based studies do not acknowledge its importance enough.

A prominent example of an evidently sex-biased cancer type is melanoma. Melanoma is a very aggressive and heterogeneous skin cancer that tends to spread to other parts of the body [3]. It is the fifth most common cancer among men and the sixth among women. Female overall survival rate advantage has been continuously confirmed for melanoma throughout the years. More specifically, after adjusting for other covariates, sex is known to be an independent prognostic factor [4,5,6]. The incidence rate of melanoma is 1.5 times higher in males than in females, and mortality due to melanoma in males is twice higher with respect to females [7,8]. Males were shown to harbor unfavorable primary tumor characteristics, such as age, histological subtype, ulceration, Breslow thickness, mitotic rate, vascular invasion, and recurrence. These factors were shown to independently predict poor outcomes in melanoma patients and lower the survival rate, reinforcing the importance of sex and sexual dimorphism in melanoma [9]. In addition to sex, stage is also a very important stratifying factor in clinical and prognostic settings. The stage of cancer at diagnosis directly affects the survival of the patient and the gene expression profile of the lesion. However, it has been found that the influence of sex on survival is limited to melanomas diagnosed at early stages. No consistent difference in survival is observed between female and male metastatic melanoma patients [10].

Even though the effects of sex on melanoma have recently started to be studied extensively [11,12], they were not as extensively focused on the aspect of gene expression. Despite melanoma and other cancer gene expression datasets being ample, they generally do not apply sex and stage stratifications in their analyses. One study that explored the sex bias in gene expression of various human and mouse tissues reported that gene expression variability is generally lower in females than males [13]. Another, more recent study analyzed the effect of sex on gene expression across various human tissues [14]. Incorporating both gene expression profiling and its variability in sex- and stage-stratified cancer studies is important for obtaining more reliable results.

In this study, through available online datasets of melanoma patients, we investigated the role of sex and stage in this disease. More specifically, we analyzed the gene expression profiles and variability differences. In addition, we explored the overall survival differences between sexes and stages as well as gene expression profiles associated with overall survival.

## 2. Materials and Methods

### 2.1. Datasets

#### 2.1.1. LMC

In order to access the Leeds Melanoma Cohort (LMC) primary melanoma transcriptomic dataset (study ID EGAS00001002922) with all the other relevant data, an agreement was signed between the University of Leeds and Fondazione Edo ed Elvo Tempia in Biella, Italy. A total of 695 patients were extracted, of which 380 were females (320 stage I, II and 50 stage III) and 315 males (268 stage I, II and 47 stage III). For the subsequent analysis, the quantile normalized gene expression matrix was used. 

#### 2.1.2. TCGA

Gene expression and other relevant data of The Cancer Genome Atlas-SKCM Cohort (TCGA-SKCM) were downloaded from the cBioPortal (www.cbioportal.org (accessed on 25 September 2021)). Any stage IV and metastatic melanomas were excluded from the analysis giving a total of 94 samples (67 stage I, II patients: 27 females and 40 males; 27 stage III samples: 13 females and 14 males). The Deseq2 log2 transformed gene expression values were used for all analyses except class comparison. The class comparison of the TCGA dataset was performed on the raw counts as explained below.

#### 2.1.3. GSE53118

The GSE53118 dataset, which was downloaded from the Gene Expression Omnibus database (https://www.ncbi.nlm.nih.gov/geo/ (accessed on 15 September 2021)), has a total of 79 stage III melanoma samples, of which 29 are females and 50 males.

#### 2.1.4. Nevi Samples

Two nevi datasets, GSE46517 (4 female and 5 male samples) and GSE3189 (12 female and 6 male samples), were also downloaded from the Gene Expression Omnibus database (https://www.ncbi.nlm.nih.gov/geo/ (accessed on 17 September 2021)). The two datasets were merged and batch effects removed, using the “removeBatchEffect” function in the “limma” package [15], to obtain the final nevi dataset. This yielded a total of 16 female and 11 male samples.

An additional nevi dataset, E-MTAB-1862, consisting of 6 female and 5 male samples, was individually analyzed to validate the results of the merged nevi dataset. Due to platform differences, this dataset was not merged to the previous two, but the common ID probes were used to compare results.

#### 2.1.5. GTEx

The normal skin gene expression data (Gene read counts from RNA-Seq) were obtained from the GTEx portal (https://gtexportal.org/home/ (accessed on 25 September 2021)). A total of 590 samples were extracted: 253 (83 females and 170 males) for non-sun-exposed skin and 337 (117 females and 220 males) for sun-exposed skin. The Deseq2 log2 normalized gene expression values were used for all analyses, except for class comparison. The class comparison of the GTEx dataset was performed on the raw counts as explained below.

### 2.2. Gene Set Enrichement Analysis (GSEA)

Biological differences between stratified groups were evaluated using GSEA. For this analysis, genes were not separated into autosomal and sex chromosomes. We focused on Hallmark (H) and ontology gene sets (C5). Analysis was performed on females compared to males at stage I, II, females compared to males at stage III, females stage I, II compared to stage III, and males stage I, II compared to stage III. For each analysis, the nominal *p*-value (NOM) and normalized enrichment scored (NES) are reported.

### 2.3. Statistical Analysis

All statistical and computational analyses were performed within the R statistical environment [16]. Unless specified otherwise, the significance cut-off for the Benjamini–Hochberg adjusted *p*-value for all relevant analyses was 0.05. Analyses were performed on autosomal and sex chromosomes genes separately.

Kaplan–Meier survival curves were generated and compared between sexes/stages, using the “survfit” function in the “survival” package [17]. Multivariate penalized Cox regression analysis was performed using the “cv.glmnet” function from the “glmnet” package [18]. A custom penalty parameter was set at 0.6×lambda.min, below which the genes were selected. The univariate Cox regression was performed using the “coxph” and “surv” functions from the “survival” package. The *p*-values were corrected for multiple testing using the Benjamini–Hochberg method. Gene variability was analyzed on all datasets based on the coefficient of variation (CV) statistic.

Comparison of CVs between the sexes (stratified by stages) was performed using the paired Wilcoxon two-sample test using the “wilcoxon.test” function. The CV of each gene within each group of interest was calculated and then log2 transformed. The z-score was then calculated on the log2 CV values for each group. 

Differential gene expression analysis between sexes (stratified by stages) was performed using the “limma” [15] and “Deseq2” [19] packages on datasets based on the microarray and RNA-Seq platforms, respectively. To correct the *p*-values for multiple testing, the Benjamini–Hochberg method was used. The log-fold changes were calculated between the analyzed groups.

To estimate the proportion of different cell types within the analyzed datasets, the complete gene expression data were processed using the “EPIC” function from the “EPIC” package [20]. The eight-cell type outputs of EPIC analysis are the B-cells, CAFs, CD4 lymphocytes, CD8 lymphocytes, endothelial cells, macrophages, NK cells, and other cells. Gene ontology analysis on gene sets of interest was performed using the DAVID bioinformatic tool (www.david.ncifcrf.org (accessed on 25 September 2021)). For a process to be considered significantly enriched, a nominal *p*-value cut-off of <0.01 was set. The default gene background offered by DAVID was used in all analyses. 

A set of genes related to melanoma or genes involved in skin development was obtained from the Molecular Signatures Database v7.5.1 (MSigDB), Human_NCBI_Gene_ID [21,22]. CV and gene expression were compared between the groups of interest on the retrieved sets of genes. More specifically, for each gene within a group, the CV and gene expression were calculated, and then the set of CVs or gene expression profiles were compared between sex/stage groups by utilizing the paired Wilcoxon two-sample test.

## 3. Results

### 3.1. Datasets and Samples

A total of seven different transcriptomic profiling datasets were analyzed: LMC, GSE53118, TCGA, GTEx (non-sun-exposed skin and sun-exposed skin), and Nevi samples. One Nevi dataset was obtained by merging two GEO datasets (GSE46517 and GSE3189), while the second dataset (E-MTAB-1862) was analyzed individually. Three of the datasets are based on NGS platforms, while the other four were obtained from microarray platforms (two Illumina, two Affymetrix). When the samples in the melanoma datasets were separated according to the stage, stages I and II were combined in one category, referred to as stage I, II in the article. The probes or the ENSEMBL gene IDs, from now on referred to as genes, of each dataset were separated into two groups according to their chromosomal location: autosomal and sex chromosomes. Details on the melanoma and non-melanoma datasets can be seen in Table 1 and Table 2, respectively.

### 3.2. Survival Analysis

**Figure 1:**
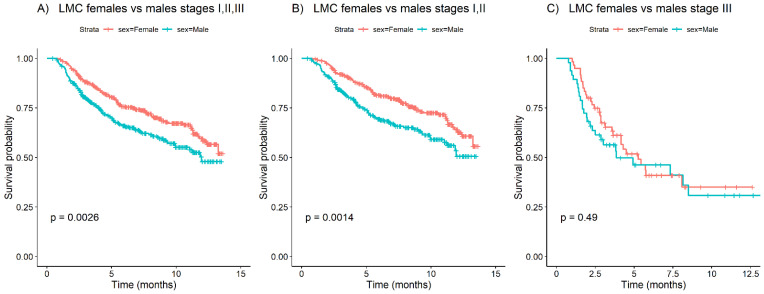
Kaplan–Meier survival curves with the log-rank *p*-value test between females and males of the LMC dataset on: (**A**) stages I, II, and III combined; (**B**) stage I, II; and (**C**) stage III.

Due to the substantially larger sample size compared to the other datasets and due to the availability of complete follow-up information, the LMC dataset was the most suitable for survival analysis. For stages I, II, and III combined, a significantly better overall survival was seen in females (Figure 1), with a log-rank test *p*-value of 0.0026. To further investigate the effect of the stage on overall survival, stages I and II were separated from stage III. Kaplan–Meier curves showed significantly better survival in females vs. males in stages I and II (log-rank test *p*-value = 0.0014). However, no significant difference in survival between sexes was observed in stage III (log-rank test *p*-value = 0.49). No significant differences in survival between sexes in any stage category computed on the other datasets were found (Supplementary Figures S1 and S2).

Univariate Cox regression analysis applied to the LMC dataset, without stratifying for stages, revealed that the genes associated with survival, either autosomal or sex chromosome genes, were more abundant and had higher statistical power within females than in males (Table S1). The same applied when only stage I, II samples were considered. Within the 21 sex chromosome genes associated with survival in stage I, II females, six were annotated as “X chromosome inactivation escape” genes. Univariate Cox analysis on the other dataset subgroups resulted in no significant genes.

Multivariate Cox regression analysis performed on the three melanoma datasets, on both autosomal and sex chromosome genes, confirmed that the genes associated with survival are different between females and males. For instance, within the LMC dataset, no common genes associated with survival were observed between female and male melanoma samples of stage I, II or of stage III. Furthermore, slightly more genes positively associated with survival were observed in stage I, II females compared to males. For both autosomal and sex chromosome genes, no common genes were found between sexes in the stratified stage groups in TCGA and GSE53118 datasets. The number of genes associated with survival in the two sexes, stratified by stage within the three datasets, can be seen in Table 3.

Functional enrichment analysis on genes obtained by the multivariate Cox regression of level 5 biological processes (BP5) of the gene ontology performed utilizing DAVID on the autosomal genes associated with survival revealed numerous interesting biological processes. Sixteen biological processes were significantly overrepresented within the 38 genes associated with better survival in stage I, II female melanomas. Interestingly, 14 of them are involved in the defense and immune response, and two of them were related to the cytokine-response process. On the other hand, the 28 genes associated with worse survival in females stage I, II were enriched in cell cycle and epigenetic regulation processes. No significant biological processes were overrepresented within the 25 genes correlated to better survival for males stages I, II. However, the 32 genes correlated with worse survival were significantly enriched in cell adhesion and epidermal growth factor signaling (Tables S2–S4).

Among analyzed sex chromosome genes associated with either better or worse survival in stage I, II, three biological processes were overrepresented in total: cellular chemical homeostasis for females and regulation of RNA metabolic process and synaptic vesicle maturation for males (Tables S5–S7). No significant processes were retrieved by DAVID for any of the stage III groups, neither for autosomal nor for sex chromosome genes.

### 3.3. Gene Expression Variability

Considering the importance of gene expression variability between sexes in literature and its association with tumor aggressiveness, we wanted to investigate whether sex differences in the number of survival-associated genes could be ascribed to differences in gene expression variability. In all datasets, the CV was calculated for each gene. For each stage group, a paired Wilcoxon two-sample test on the set of calculated CVs was performed between females and males. Differences in CV between sexes stratified by stage were analyzed in the datasets. Significantly different CV on autosomal chromosome genes was observed between melanoma samples of males and females in both stage groups (stage I, II and stage III), in all datasets. A significantly lower CV in females was observed in the Nevi (in both analyzed datasets) and Gtex (non-sun-exposed and sun-exposed skin) datasets. Moreover, significantly different CV in sex chromosome genes was observed in all mentioned datasets. Details can be seen in Table 4.

Interestingly, stage I, II female melanoma samples had lower average CV than males both in LMC and TCGA, whereas the opposite was observed in stage III samples. This observation was confirmed in the GSE53118 dataset, which contains stage III samples only. Additionally, in both LMC and TCGA datasets, a significant difference in gene variability was observed between stages I, II and III in both females and males.

**Figure 2:**
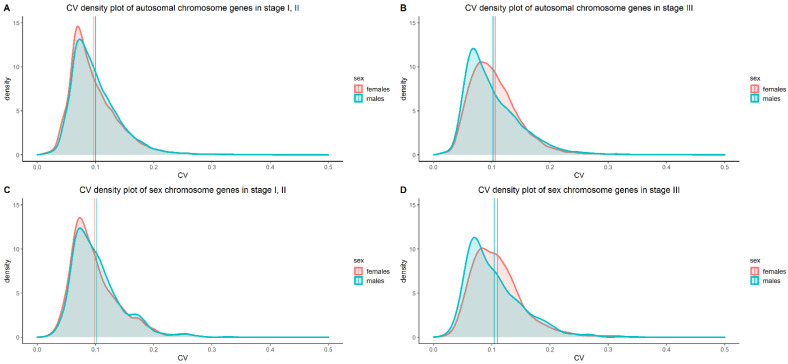
CV density plots, of the LMC gene expression data, on the sexes was created for: (**A**) autosomal chromosome genes in stage I, II; (**B**) autosomal chromosome genes in stage III; (**C**) sex chromosome genes in stage I, II, and (**D**) sex chromosome genes in stage III. The vertical lines on the x-axes represent the average CV of each sex for each analyzed subcategory.

Density plots of the CV in stage I, II and stage III samples, obtained from the LMC dataset, in males and females can be seen in Figure 2. The density plots of other datasets can be found in Figures S3–S6.

Gene set enrichment analysis (GSEA) was applied to uncover stage specific biological differences for each sex. Analysis of females stage I, II compared to females stage III revealed two enriched immune-related processes in stage I, II, GOBP_natural_killer_cell_activation_involved_in_immune_response (NES = 1.598, NOM *p*-value = 0.04) and GOBP_toll_like_receptor_3_signaling_pathway (NES = 1.579, NOM *p*-value = 0.03). In stage III females, the process HP_chromosome_breakage (NES = −1.768, NOM *p*-value = 0.008) was significantly overrepresented. On the other hand, GSEA did not show any enriched pathways for stage III compared to stage I, II males. However, in stage I, II males vs females, there were multiple significantly enriched biological hallmarks such as HALLMARK_KRAS_signaling_up (NES = 1.610, NOM *p*-value = 0.01), HALLMARK_inflammatory_response (NES = 1.678, NOM *p*-value = 0.02), and GOBP_response_to_interleukin_6 (NES = 1.772, NOM *p*-value = 0.002). Stage III male vs female melanomas were significantly enriched in several DNA polymerase pathways such as GOMF_DNA_polymerase_activity (NES = −1.800, NOM *p*-value = 0) and telomere lengthening related pathways such as GOCC_telomerase_holoenzyme_complex (NES = −1.825, NOM *p*-value = 0.006).

**Figure 3:**
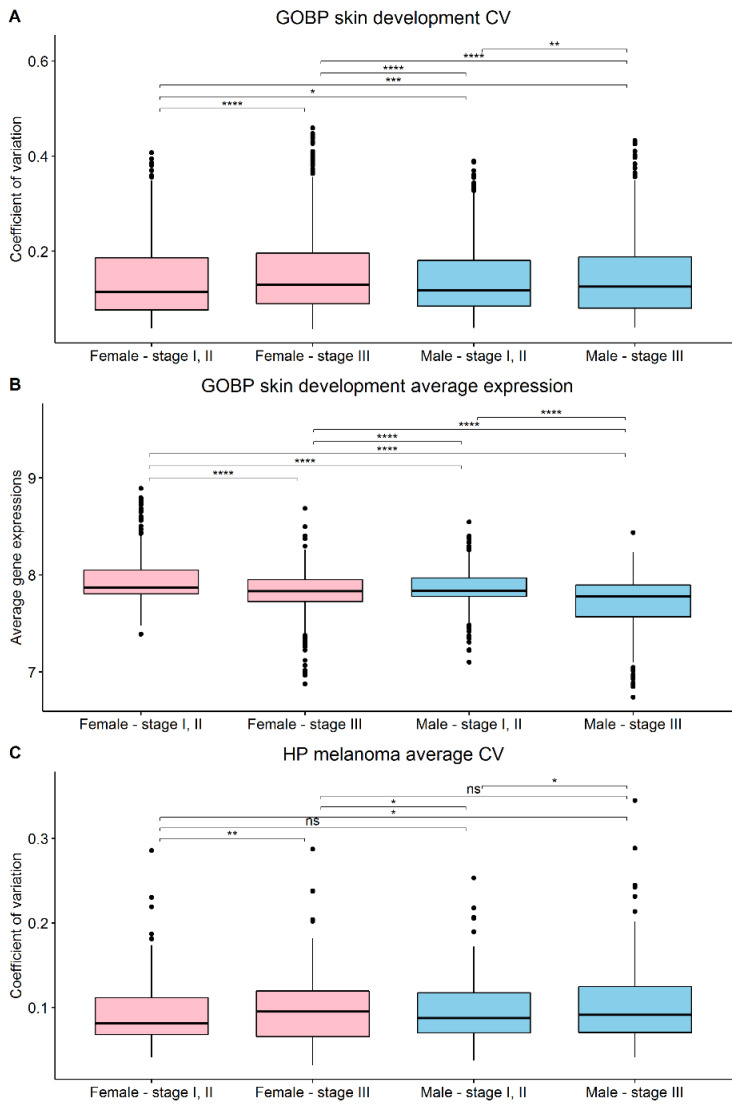
Boxplots on the sex/stage stratifications were constructed on (**A**) CVs of the gene signature for GOBP_skin_development, (**B**) gene expression averages of the gene signature for GOBP_skin_development, and (**C**) CVs of the gene signature for HP_melanoma. ns = not significant, * *p* < 0.05, ** *p* < 0.01, *** *p* < 0.001, **** *p* < 0.0001.

Specific processes in melanoma or skin development consisting of defined gene signatures were further considered. More specifically, differences in gene expression CVs between sexes or stages were analyzed within the LMC dataset. GOBP_skin_development gene signature from MSigDB was observed to have significantly different CV between females and males in stage I, II, as well as in stage III (Figure 3A). Furthermore, a significant difference was observed between stages I, II and stage III within the same sex. Another gene signature retrieved from MSigDB, called HP_melanoma, was found to have significantly different CVs between the sexes of the same stage group but no statistically significant difference between stage groups within the same sex (Figure 3C).

Based on the z-score calculated on log-transformed average CV values from the LMC dataset, genes with a z-score larger than 2 (highly unstable genes) or smaller than −2 (highly stable genes) were selected for further functional enrichment analysis. For stage I, II, there were 593 highly variable unique genes found in females, while 578 unique genes were found in males, and 491 of them were common between the sexes and were mainly enriched in skin-related processes based on DAVID GO analysis. The 102 genes highly variable only in females were found to be mainly involved in immune-system-related processes, while the remaining processes were skin related. No significant biological processes were observed for the 87 highly variable genes in males. For stage III, the common highly variable genes were enriched mostly in skin-related processes. Moreover, genes highly variable only in females were found to be significantly enriched in only two processes, while highly variable genes in males were mainly found to be involved in nervous system developmental processes. The gene ontology results of the groups of interest on the highly variable genes can be found in Tables S8–S13.

Concerning the stable genes, for stages I, II, 535 unique, highly stable genes were found in females, while 519 unique genes were found in males. In total, 416 of those genes were common between the sexes and were enriched in several RNA processing and post-transcriptional regulation processes, and 119 genes that were highly stable exclusively in females were found to be mainly enriched in metabolic process, protein localization, and RNA processing. On the other hand, the 103 genes highly stable only in males were found to be associated with metabolic and cell-cycle processes. In stage III, there were 610 highly stable unique genes in females and 409 in males. In total, 291 were common between the two sexes and were enriched in various RNA metabolic processes as well as protein synthesis and localization. More than 100 highly heterogeneous processes were significantly overrepresented within female-specific genes (319), making a general interpretation highly challenging. As for males (118 genes), only 11 processes were found to be significant, most of which were related to protein metabolic modification or DNA repair. The gene ontology results of the groups of interest on the stable genes can be found in Tables S14–S19.

**Figure 4:**
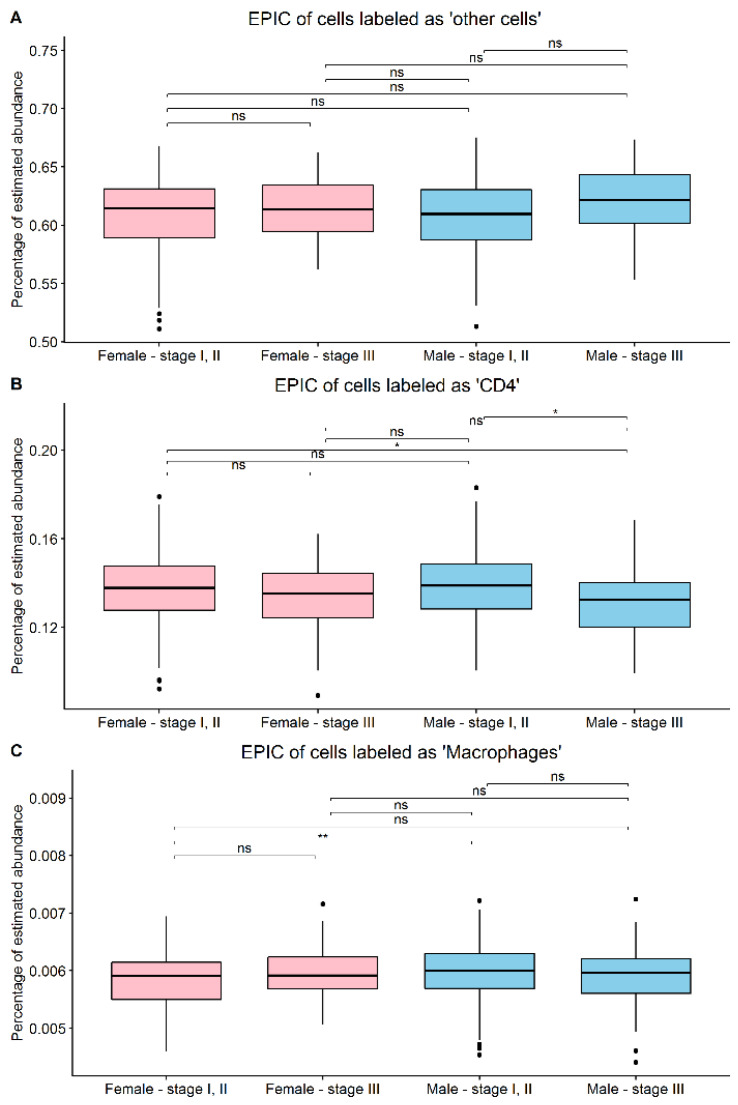
Estimate proportion of immune and cancer cells (EPIC) was performed on the sex/stage subgroups. The estimate was done on cells labeled as (**A**) “other cells”—representing for the most part cancer cells, (**B**) CD4 T cells, and (**C**) macrophages. ns = not significant, * *p* < 0.05, ** *p* < 0.01.

In order to test whether higher gene expression variability corresponds to higher enrichment of different cell types within samples, we applied EPIC deconvolution analysis to all the samples of the LMC dataset (only the cells with a significant difference within sex and stage or cells of interest are shown). Differences in the proportion of cell types were then analyzed between the stratified groups. The cell type with the highest proportion was labeled as “other cells”, which are mostly cancer cells. No significant difference in cell proportion of “other cells” was observed between the stratified groups. This indicates that the gene expression variation differences observed between the groups were not a by-product of the tissue sampling. Furthermore, CD4 lymphocytes were observed to have a significantly different proportion between females stage I, II and males stage III as well as between males stage I, II and males stage III. In addition, the cell-type macrophages were found to be significantly more abundant in males stage I, II with respect to females stage I, II tissue samples (Figure 4). Further investigation regarding the biomarkers of M1 and M2 macrophages revealed that four M2-related genes (CD163, MSR1, IL2RA, and CCL18) were found to be significantly (raw *p*-value < 0.01) higher in males stage I, II than females stage I, II (Additional file 2).

### 3.4. Class Comparison Analysis

Differential expression between the sexes was analyzed on the three melanoma datasets as well as on the Nevi and normal skin datasets (Additional file 1). Details on the number of overexpressed genes in each of the sexes can be seen in Table 5. Interestingly, the number of differentially expressed genes between sexes showed a decreasing trend from normal skin to nevi and from early-stage to late-stage melanomas. Moreover, unlike the normal skin dataset and the melanoma stage I, II groups, very few differentially expressed genes were observed between sexes within the NEVI datasets.

The overexpressed genes within each sex stratified by stage were selected for gene ontology analysis by DAVID. Gene ontology analysis on autosomal chromosome overexpressed genes within the groups of interest from the LMC dataset showed significant enrichment for the fat cell differentiation process in females stage I, II. On the other hand, overexpressed genes in males stage I, II were significantly enriched, mainly in immune response and protein regulation (Tables S20 and S21). 

The gene expression analysis of the specific GOBP_skin_development gene signature revealed a statistically significant difference between females and males in stage I, II as well as in stage III (Figure 3B). Furthermore, a significant difference was observed between stage I, II and stage III within the same sex.

**Figure 5:**
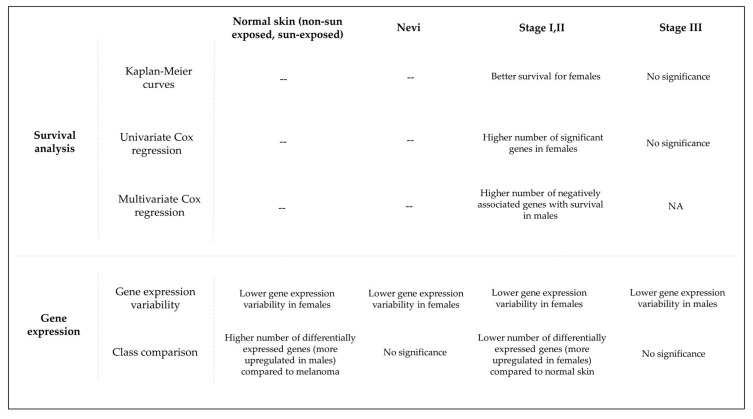
Summary of the methodologies and results of this study.

For a clearer presentation of the methodology and the results, a summary of the study is shown in Figure 5.

## 4. Discussion

In the present work, we analyzed gene expression datasets of stage I, II, and III melanomas, common acquired nevi, and normal skin in order to emphasize the importance of sex and stage categorization in early-stage melanoma.

It is well known that prognosis is generally worse for males than for females, and this may depend in part on a later stage diagnosis and in part on a different behavior of the lesions [23]. Statistical analyses of melanoma transcriptomic profiles rarely take both sex and stage into account, especially when it comes to the identification of prognostic biomarkers [24].

In our study, Kaplan–Meier curves stratified by sex obtained from the analyzed datasets confirmed that females have better overall survival in melanoma than males when stages I, II, and III are taken together. In stage-specific subsets, this observation only holds true for stages I and II, either taken separately or combined. In none of the analyzed datasets was a significant sex difference observed in overall survival for stage III melanomas. Similar findings were previously observed by two other studies [25,26].

Univariate Cox regression on overall survival highlighted that when both sexes are considered together, most of the genes associated with survival in early stages derive from female samples. Among those genes, 30% of the sex chromosome ones are involved in the escape from X chromosome inactivation, a protective mechanism already found in previous studies [27,28]. Moreover, the number of genes associated with survival differs both between the same stage in females and males and between females and males of different stages. The difference is reflected not only in the number but also in the functions in which those genes are involved. For example, gene ontology analysis on genes obtained by penalized Cox regression revealed that a high number of immune-related processes were overrepresented within the genes protective for female stage I, II melanomas. This was, however, not observed neither in stage III female nor in stage I, II male melanoma samples.

In contrast, when the characteristics of the highly variable genes were analyzed, a higher concordance was observed between sexes and stages. More specifically, both in stage I, II and in stage III, the majority of genes with the highest expression variability found within males and females were common between the two sexes and were significantly enriched mainly in skin-related processes. Among sex-specific processes, we highlighted the enrichment of nervous system development within highly variable genes in stage III male melanomas, which may be linked to the neural crest embryonic origin of melanocytes and their destabilization/transformation [29].

Interestingly, all tested melanoma datasets were in concordance regarding the gene expression variability difference observed within the sex and stage subcategories. More specifically, a lower gene expression variability in female samples was confirmed in stage I, II melanomas from the LMC and TCGA datasets. On the other hand, gene expression was more variable in stage III female samples in the LMC, TCGA, and GSE53118 datasets.

Normal skin and common acquired nevi samples were also analyzed to evaluate any difference in gene expression variability between the sexes. Indeed, in both datasets, female samples showed a lower gene expression variability, as also observed by Itoh and Arnold 2015 [13], pointing out that, when it comes to gene variability, normal skin and nevi samples are more similar to the early stages of this disease.

We, therefore, hypothesize that the higher number of genes associated with survival in females compared to male stage I, II samples may be explained by the larger gene expression heterogeneity in males. Hence, the same explanation can be applied to the fact that no genes significantly associated with survival were obtained in stage III samples, neither for females nor males, as in both sexes, gene expression was found to be significantly more heterogeneous in stage III than in stage I, II samples. This goes in line with the well-reported positive correlation of melanoma heterogeneity with stage progression. Consequently, increased genetic instability in the tumor causes transcriptomic and proteomic diversity, which in turn allows microenvironment-driven or cell-intrinsic phenotype-switching, i.e., reversible switching between different phenotypes of proliferative and invasive potentials [30,31].

EPIC analysis on the LMC dataset showed that the differences observed in gene expression variability were not attributable to differences in the sampling of the tissues, as the cells labeled by EPIC as “other cells”, generally considered to be cancer cells, did not have any significant differences between the groups. Interestingly, among the tumor microenvironment cell types analyzed by EPIC, only macrophages were found to be significantly higher in male compared to female stage I, II samples. As a spectrum of macrophages is known to have a pro-cancer activity [32], the observed EPIC results, in addition to the M2 biomarkers [33] being overexpressed in males, suggest a higher abundance of potentially harmful macrophages (M2) in the microenvironment of male stage I, II melanomas. In addition, as CD4 is known to have anti-tumor activity in melanoma [34], observing a significantly lower proportion of CD4 T cells in males stage III could possibly explain the significantly worse overall survival observed in this group compared to stage I, II females and males, as well as the worse overall survival trend compared to stage III females.

Class comparison of gene expression between sexes showed a higher number of differentially expressed genes between females and males in stage I, II compared to stage III. Moreover, the number of genes that were differentially expressed between females and males of both sun- and non-sun-exposed normal skin was substantially higher than the number of genes obtained from the melanoma datasets, indicating that sex differences are more pronounced in non-malignant than in malignant tissue, which is supported by the finding that sex differences decrease as the stages progress [10]. Among the under-expressed genes in stage I, II males located on the X chromosome, nine that are known tumor suppressors in melanoma and/or other cancers (Additional file 1). Despite no differentially expressed genes found in stage III between the sexes, Figure 3B shows that skin development genes are significantly under-expressed in male stage III patients, which might be explained by a possible occurrence of higher cell dedifferentiation, which therefore results in a more aggressive phenotype.

Through GSEA analysis, some important stage differences were observed for each sex. Two immune-related pathways were overrepresented in stage I, II compared to stage III female samples. This might, in part, explain the overall better survival rate observed in stage I, II. In stage III females, the enriched chromosome breakage [35] pathway is in accordance with tumor progression, genetic instability, and gene expression variability differences observed. Genes overexpressed in males stage I, II were enriched in RAS signaling and inflammatory pathways, which are early events driving tumor development and progression [36,37]. In addition, pathways related to key tumor proliferation enzymes, telomerase, and DNA polymerase were significantly overrepresented in stage III males. Interestingly, GSEA stage differences can be, to an extent, compared to gene ontology analysis done on genes associated with better survival from the multivariate Cox regression analysis, where genes in stage I, II females were associated with immune-related processes. Based on the results presented in this as well as other previous studies [38], immune-related processes are a big part of sexual dimorphism. However, interpretation of the role of the immune system and its differences between sexes, especially in early-stage melanomas, is not always linear.

## 5. Conclusions

In this study, gene expression datasets of early-stage melanomas, nevi, and normal skin were analyzed. Clear differences were found between sexes and stages in terms of survival-associated genes, transcriptomic profiles, and variability, highlighting the sexual dimorphism reflected in gene expression. Finally, the importance of taking sex and stage into account in future transcriptomic or biomarker melanoma studies is emphasized.

## Figures and Tables

**Table 1:** Detailed characteristics of the analyzed melanoma datasets (st = stage).

	Leeds Melanoma Cohort (LMC)				GSE53118				TCGA			
Sex	F	M	F	M	F	M	F	M	F	M	F	M
Stage	I, II	I, II	III	III	I, II	I, II	III	III	I, II	I, II	III	III
Number of patients	320	268	60	47	0	0	29	50	27	40	13	14
Mean age	52.87	58.42	56.41	59.54	0	0	59.79	59.42	66.56	66.35	65.31	56.57
<50 years old (%)	37.81%	23.13%	31.67%	18.75%	0	0	24%	18%	11%	13%	23%	21%
>50 years old (%)	62.19%	76.87%	68.33%	81.25%	0	0	76%	82%	89%	88%	77%	79%
# of dead patients	26%	34%	52%	55%	0	0	59%	60%	30%	13%	15%	64%
Platform	Illumina HT 12.4 (GPL28098-111924)				Illumina WG-6 v3.0 (GPL6884-11607)				Illumina HiSeq 2000 RNA-seq			
Autosomal chr. IDs	28,019				25,004				16,409			
Sex chr. IDs	1227				1014				663			

**Table 2:** Detailed characteristics of the analyzed non-melanoma datasets.

Datasets	Females	Males	Platform	No. of Autosomal chr. IDs	No. of Sex chr. IDs
GTEx non-sun-exposed	83	170	Illumina TrueSeq RNA-seq	25239	0
GTEx sun-exposed	117	220	Illumina TrueSeq RNA-seq	25193	0
NEVI (E-MTAB-1862)	6	5	Affymetrix HG-U133_Plus_2	21430	761
NEVI (GSE46517+GSE3189)	16	11	Affymetrix HG-U133A (GPL96)	21430	761

**Table 3:** Multivariate Cox regression analysis on the LMC, TCGA, and GSE53118 melanoma dataset. Analysis was performed on autosomal and sex chromosome genes on females and males together for stages I, II, and III combined, stage I, II, and stage III. The same analyses were performed for the stage categories by separating the sexes. The significant genes obtained within each sex and stage subcategory are assigned with a “+” if associated with better overall survival and with a “−” if associated with worse overall survival.

		Leeds Melanoma Cohort (LMC)		TCGA		GSE53118	
Stage	Sex	Autosomal chr. Genes	Sex chr. Genes	Autosomal chr. Genes	Sex chr. Genes	Autosomal chr. Genes	Sex chr. Genes
st I, II, III	Female–Male	72	99	20	19	N/A	N/A
	Female	152 (+85|−67)	51 (+21|−30)	10 (+5|−5)	15 (+5|−10)	N/A	N/A
	Male	69 (+23|−46)	20 (+6|−14)	14 (+4|−10)	12 (+2|−10)	N/A	N/A
st I, II	Female–Male	108	81	21	17	N/A	N/A
	Female	66 (+38|−28)	65 (+25|−40)	7 (+3|−4)	7 (+3|−4)	N/A	N/A
	Male	57 (+25|−32)	67 (+28|−39)	6 (+3|−3)	7 (+2|−5)	N/A	N/A
st III	Female–Male	30	23	13	9	25	19
	Female	21 (+8|−13)	24 (+11|−13)	5 (+2|−3)	2 (+1|−1)	11 (+11|−3)	14 (+7|−7)
	Male	22 (+10|−12)	17 (+4|−13)	8 (+3 |−5)	8 (+2|−6)	21 (+11|−10)	14 (+8|−6)

**Table 4:** Average coefficients of variation on the autosomal and sex chromosome genes of the melanoma (LMC, TCGA, GSE53118) and non-melanoma (Nevi, Normal skin) datasets were calculated for both females and males in the stratified stage groups. A Wilcoxon paired test between females and males was performed on the total number of genes included in every subcategory. The paired Wilcoxon test was also performed between stages I, II and stage III within the same sex on LMC and TCGA datasets. S-E = sun-exposed.

		Autosomal Chromosome Genes—Average CV			Sex Chromosome Genes—Average CV		
Dataset	Stage	Females	Males	*Wilcoxon p-Value (Sex)*	Females	Males	*Wilcoxon p-Value (Sex)*
LMC	st I, II, III	0.09883	0.10069	*0*	0.10130	0.10290	*0*
	st I, II	0.09705	0.10009	*0*	0.09914	0.10237	*0*
	st III	0.10581	0.10238	*0*	0.10986	0.10418	*0*
	*Wilcoxon p-value (stage I, II vs. III)*	*0*	*0*	/	*0*	*0.02004*	/
TCGA	st I, II, III	0.28850	0.29157	*0.00011*	0.50386	0.48530	*0.23076*
	st I, II	0.27337	0.28902	*0*	0.47916	0.47171	*0.00043*
	st III	0.31018	0.29083	*0*	0.47514	0.46635	*0*
	*Wilcoxon p-value (stage I, II vs. III)*	*0*	*0*	/	*0*	*0.00838*	/
GSE53118	st I, II, III	N/A	N/A	*N/A*	N/A	N/A	*N/A*
	st I, II	N/A	N/A	*N/A*	N/A	N/A	*N/A*
	st III	0.04719	0.04477	*0*	0.05200	0.05295	*0.00023*
NEVI (GSE46517,GSE3189)	N/A	0.11755	0.12098	*0*	0.11868	0.12272	*0.00160*
NEVI (E-MTAB-1862)	N/A	0.04698	0.04705	*0.0002*	0.04802	0.04681	*0.06875*
GTEx non-S-E skin	N/A	0.24389	0.24849	*0*	N/A	N/A	*N/A*
GTEx S-E skin	N/A	0.24159	0.24164	*0.00495*	N/A	N/A	*N/A*

**Table 5:** Up-regulated autosomal and sex chromosome genes between females and males discovered by the class comparison analysis on all datasets. Melanoma gene expression datasets (LMC, TCGA, GSE53118) were further classified according to stage.

		Up-Regulated Autosomal Chromosome Genes		Up-Regulated Sex Chromosome Genes	
Dataset	Stage	Females	Males	Females	Males
LMC	st I, II, III	45	24	67	54
	st I, II	27	17	64	4
	st III	0	0	0	0
TCGA	st I, II, III	39	18	17	3
	st I, II	77	37	23	5
	st III	39	21	16	2
GSE53118	st I, II, III	N/A	N/A	N/A	N/A
	st I, II	N/A	N/A	N/A	N/A
	st III	0	0	5	0
NEVI (GSE53118, GSE3189)	N/A	0	0	3	0
NEVI (E-MTAB-1862)	N/A	0	1	2	0
GTEx non-S-E skin	N/A	604	939	N/A	N/A
GTEx S-E skin	N/A	924	1018	N/A	N/A

## Data Availability

GSE53118, GSE46518, and GSE3189 are available for download in the Gene expression Omnibus repository (https://www.ncbi.nlm.nih.gov/geo/ (accessed on 15 September 2021 and 17 September 2021)). TCGA is available in cBioPortal (www.cbioportal.org (accessed on 25 September 2021)) repository, and the GTEx dataset is available on the GTEx portal (https://gtexportal.org/home/ (accessed on 25 September 2021)). The Leeds Melanoma Cohort (LMC) was accessible through an agreement between the two institutions.

## Supplementary Materials

The following supporting information can be downloaded at: https://www.mdpi.com/article/10.3390/cells11071099/s1, Figure S1: Kaplan–Meier survival curves with the log-rank *p*-value test between females and males of the TCGA dataset on (A) stage I, II, and III combined, (B) stage I, II, and (C) stage III; Figure S2: Kaplan–Meier survival curves with the log-rank *p*-value test between females and males of the GSE53118 dataset on stage III; Table S1: Univariate Cox regression analysis on the three melanoma datasets. Analysis was performed on autosomal and sex chromosome genes on females and males together for stages I, II, and III combined, stage I, II, and stage III. The same analyses were performed for the stage categories by separating the sexes. Number of genes associated with better survival is labeled with a ‘+’, while the number of genes associated with worse survival is labeled with a ‘−’; Figure S3: CV density plots, of the TCGA gene expression data, on the sexes was created for (A) autosomal chromosome genes in stage I, II, (B) autosomal chromosome genes in stage III, (C) sex chromosome genes in stage I, II and (D) sex chromosome genes in stage III. The vertical lines on the x-axes represent the average CV of each sex for each analyzed subcategory; Figure S4: CV density plots, of the GSE53118 stage III gene expression data, on the sexes was created for (A) autosomal chromosome genes and (B) sex chromosome genes. The vertical lines on the x-axes represent the average CV of each sex for each analyzed subcategory; Figure S5: CV density plots, of the Nevi gene expression data, on the sexes was created for (A) autosomal chromosome genes and (B) sex chromosome genes. The vertical lines on the x-axes represent the average CV of each sex for each analyzed subcategory; Figure S6: CV density plots, of the normal skin autosomal gene expression data, on the sexes was created for (A) non-sun-exposed skin and (B) sun-exposed skin. The vertical lines on the x-axes represent the average CV of each sex for each analyzed subcategory. Table S2: Functional enrichment analysis on autosomal chromosome genes, using DAVID, on the level 5 biological processes was performed on 38 genes positively associated with survival in females stage I, II; Table S3: Functional enrichment analysis on autosomal chromosome genes, using DAVID, on the level 5 biological processes was performed on 28 genes negatively associated with survival in females stage I, II; Table S4: Functional enrichment analysis on autosomal chromosome genes, using DAVID, on the level 5 biological processes was performed on 32 genes negatively associated with survival in males stage I, II; Table S5: Functional enrichment analysis on sex chromosome genes, using DAVID, on the level 5 biological processes was performed on 25 genes positively associated with survival in females stage I, II; Table S6: Functional enrichment analysis on sex chromosome genes, using DAVID, on the level 5 biological processes was performed on 28 genes positively associated with survival in males stage I, II; Table S7: Functional enrichment analysis on sex chromosome genes, using DAVID, on the level 5 biological processes was performed on 39 genes negatively associated with survival in males stage I, II; Table S8: Functional enrichment analysis, using DAVID, on the level 5 biological processes was performed on highly variable genes common between female and male stage I, II samples, with a z-score > 2; Table S9: Functional enrichment analysis, using DAVID, on the level 5 biological processes was performed on highly variable genes found exclusively in female stage I, II samples, with a z-score > 2; Table S10: Functional enrichment analysis, using DAVID, on the level 5 biological processes was performed on highly variable genes found exclusively in male stage I, II samples, with a z-score > 2; Table S11: Functional enrichment analysis, using DAVID, on the level 5 biological processes was performed on highly variable genes common between female and male stage III samples, with a z-score > 2; Table S12: Functional enrichment analysis, using DAVID, on the level 5 biological processes was performed on highly variable genes found exclusively in female stage III samples, with a z-score > 2; Table S13: Functional enrichment analysis, using DAVID, on the level 5 biological processes was performed on highly variable genes found exclusively in male stage III samples, with a z-score > 2; Table S14: Functional enrichment analysis, using DAVID, on the level 5 biological processes was performed on highly stable genes common between female and male stage I, II samples, with a z-score < −2; Table S15: Functional enrichment analysis, using DAVID, on the level 5 biological processes was performed on highly stable genes found exclusively in female stage I, II samples, with a z-score < −2; Table S16: Functional enrichment analysis, using DAVID, on the level 5 biological processes was performed on highly stable genes found exclusively in male stage I, II samples, with a z-score < −2; Table S17: Functional enrichment analysis, using DAVID, on the level 5 biological processes was performed on highly stable genes common between female and male stage III samples, with a z-score < −2; Table S18: Functional enrichment analysis, using DAVID, on the level 5 biological processes was performed on highly stable genes found exclusively in female stage III samples, with a z-score < −2; Table S19: Functional enrichment analysis, using DAVID, on the level 5 biological processes was performed on highly stable genes found exclusively in male stage III samples, with a z-score < −2; Table S20: Functional enrichment analysis, using DAVID, on the level 5 biological processes was performed on genes significantly overexpressed in females compared to males in stage I, II; Table S21: Functional enrichment analysis, using DAVID, on the level 5 biological processes was performed on genes significantly overexpressed in males compared to females in stage I, II. Additional file 1: Identification of tumor suppressor (TS) genes; Additional file 2: The biomarkers of M1 and M2 macrophages in stage I, II* and III.
