# Supplementary figures and images for: Comprehensive Gene Expression Analysis to Identify Differences and Similarities between Sex- and Stage-Stratified Melanoma Samples
Source: Cells. 2022 Mar 24;11(7):1099. doi: 10.3390/cells11071099 (PMC8997401; doi:10.3390/cells11071099)

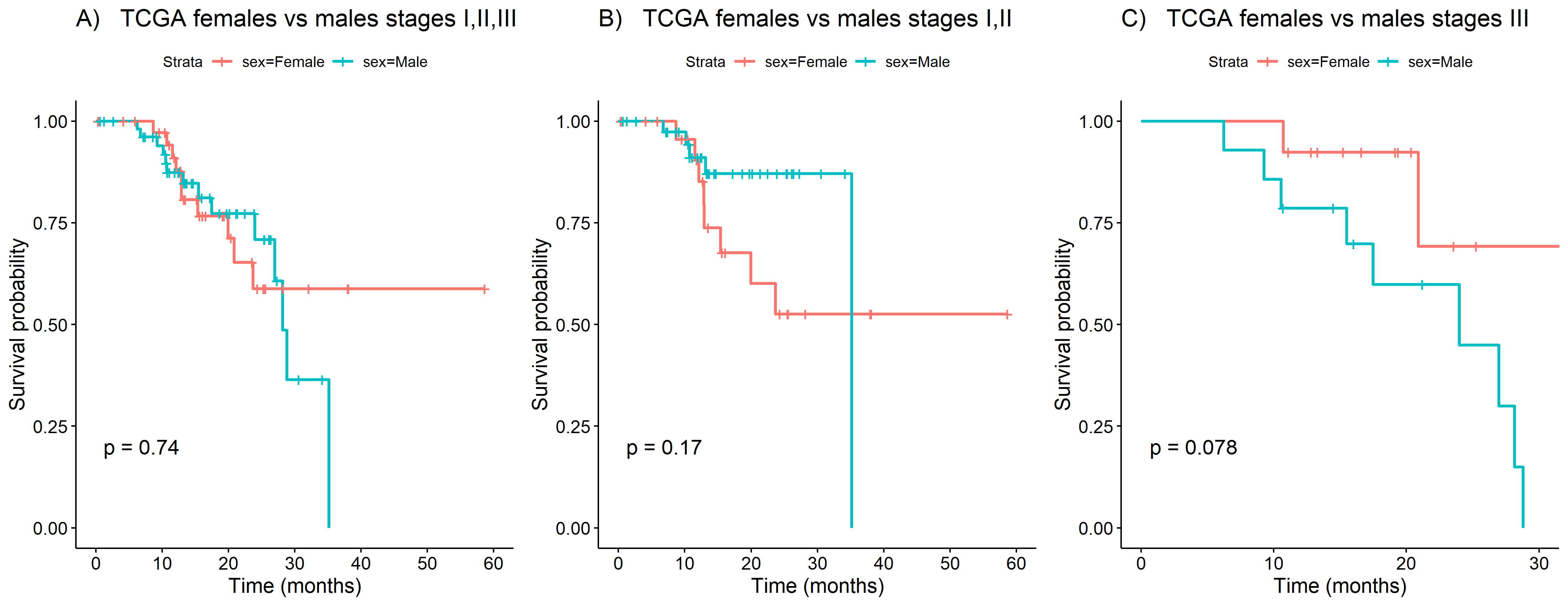

Supplement: Supplementary file 1 [file cells-11-01099-s001.zip › Figure S1.tiff]

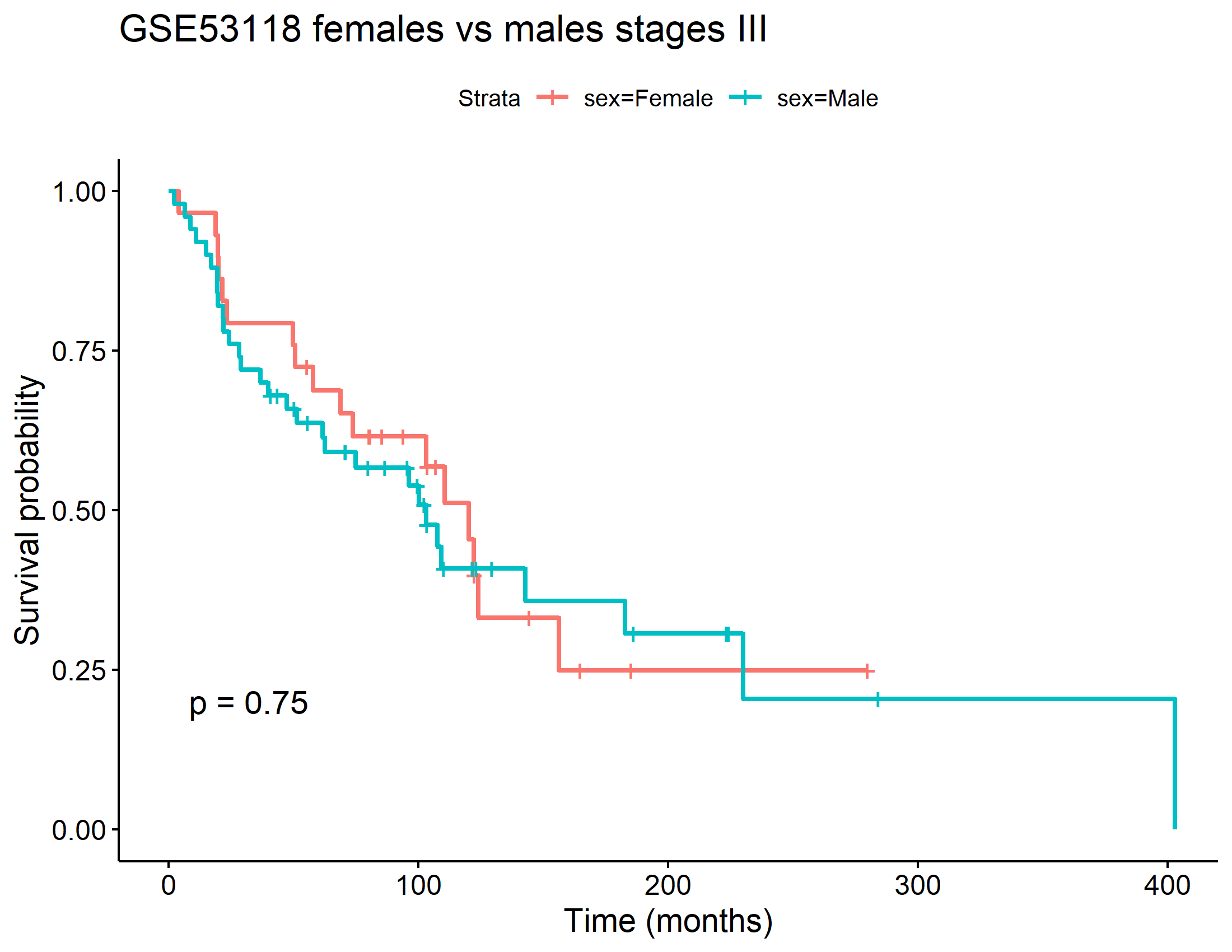

Supplement: Supplementary file 1 [file cells-11-01099-s001.zip › Figure S2.tiff]

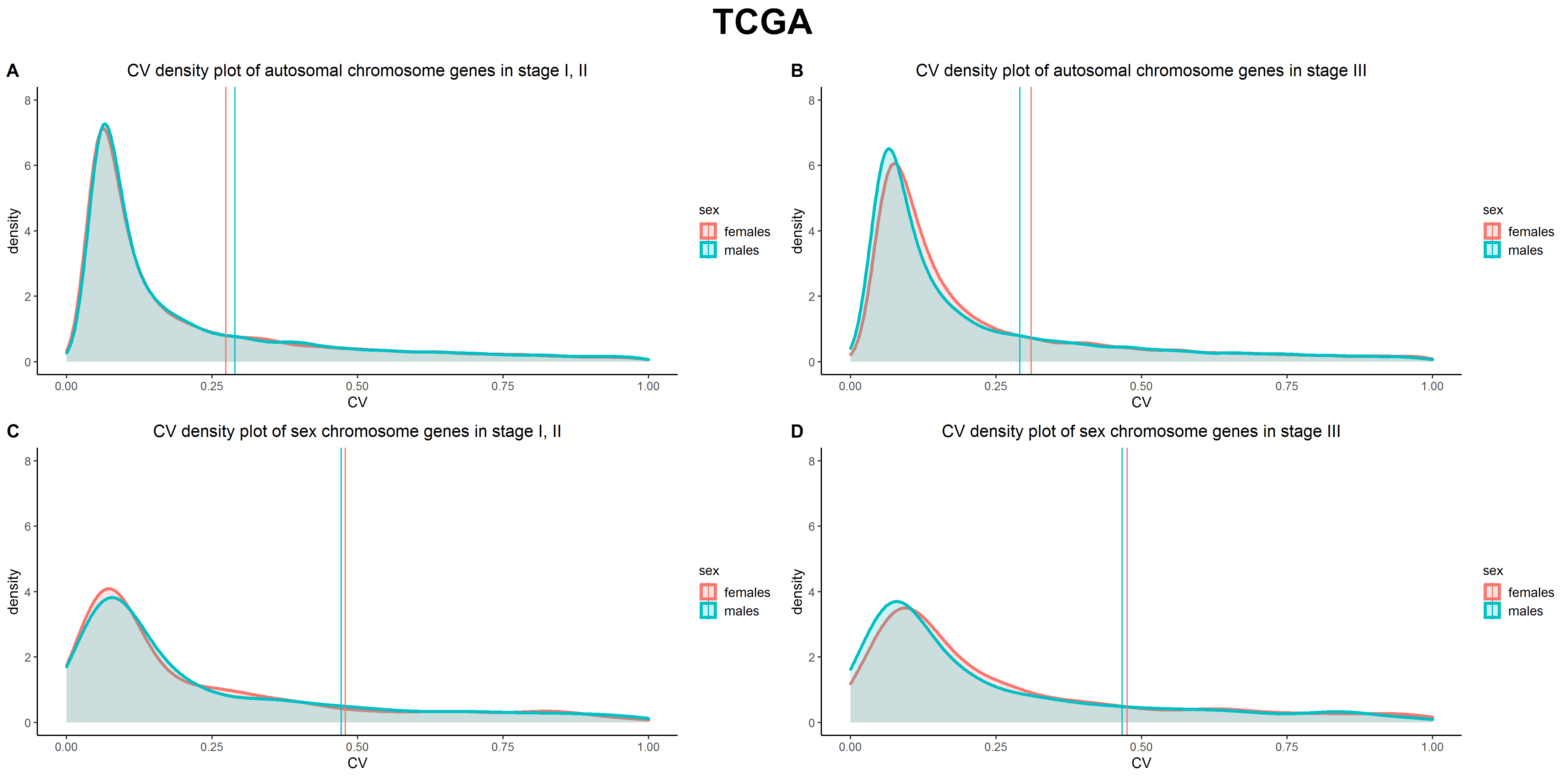

Supplement: Supplementary file 1 [file cells-11-01099-s001.zip › Figure S3.tiff]

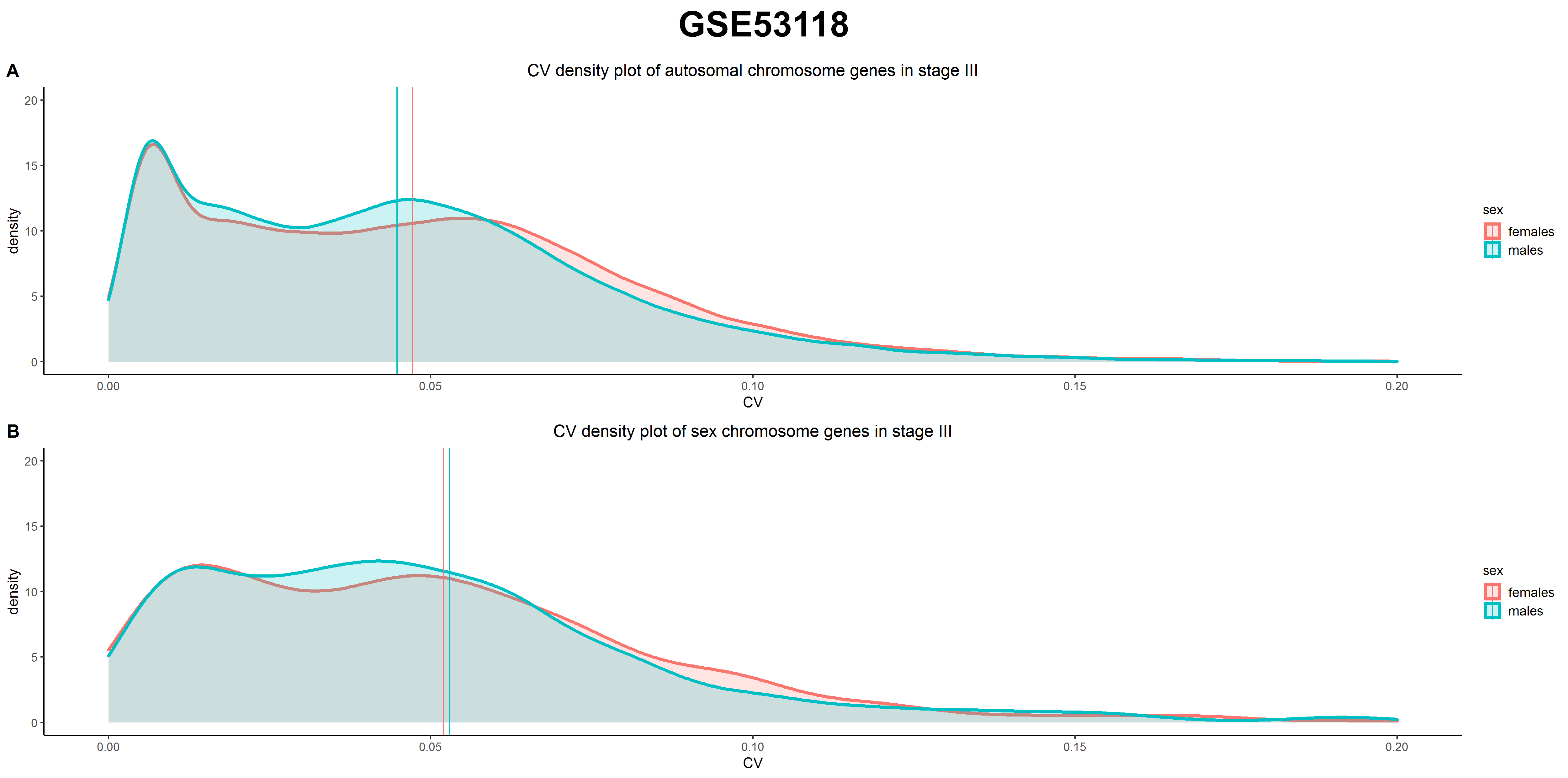

Supplement: Supplementary file 1 [file cells-11-01099-s001.zip › Figure S4.tiff]

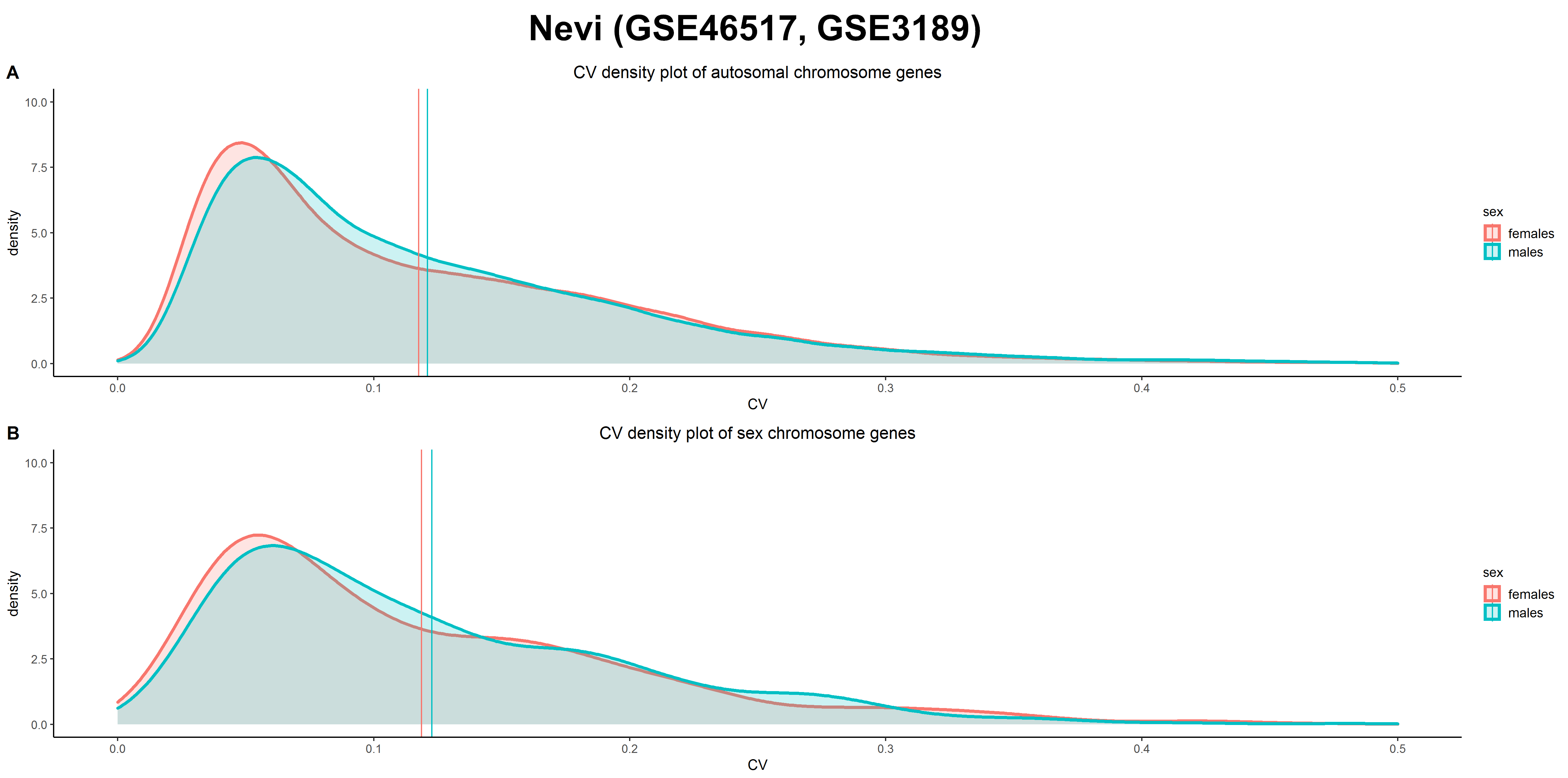

Supplement: Supplementary file 1 [file cells-11-01099-s001.zip › Figure S5.tiff]

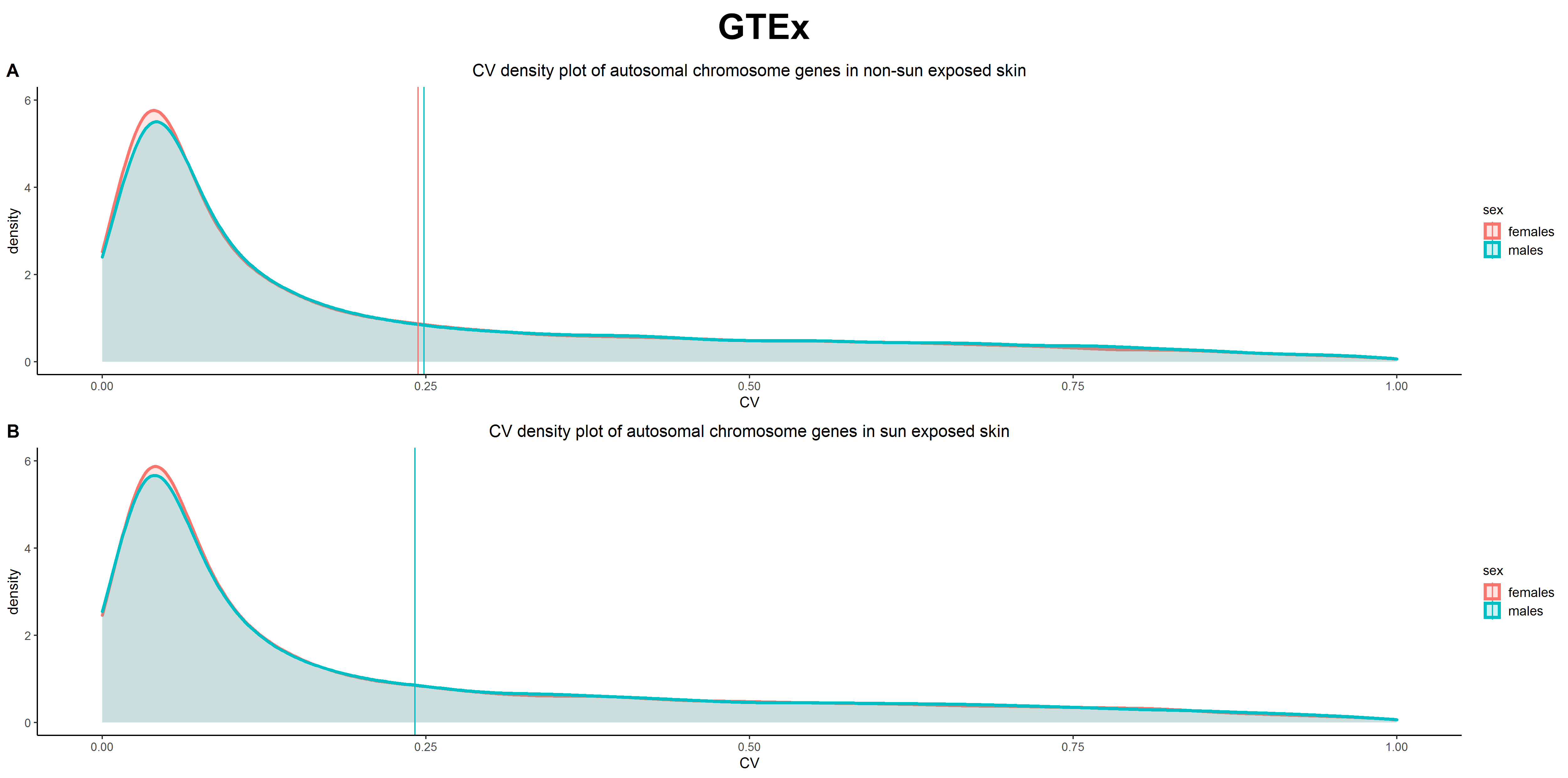

Supplement: Supplementary file 1 [file cells-11-01099-s001.zip › Figure S6.tiff]
